# Evaluation of large language models in medical examinations: A scoping review protocol

**DOI:** 10.1371/journal.pone.0347539

**Published:** 2026-04-22

**Authors:** Weiqi Wang, Baifeng Wang, Yan Zhu, Zhe Wang, Suyuan Peng

**Affiliations:** 1 College of Medical Information, Changchun University of Chinese Medicine, Changchun City, Jilin Province, China; 2 Dongzhimen Hospital, Beijing University of Chinese Medicine, Beijing, China; 3 Institute of Information on Traditional Chinese Medicine China Academy of Chinese Medical Sciences, Beijing, China; 4 Institute of Basic Medical Sciences and School of Basic Medicine, Chinese Academy of Medical Sciences and Peking Union Medical College, Beijing, China; 5 Institute for Medical Informatics, Statistics and Epidemiology, University of Leipzig, Leipzig, Germany; Western Carolina University, UNITED STATES OF AMERICA

## Abstract

**Introduction:**

Standardized medical examinations, used to assess trainee clinical competencies, provide a rigorous means to verify LLM accuracy and reliability in medical contexts. Although current evaluations use these exams to test LLMs’ clinical reasoning, significant performance variations occur across different clinical scenarios. Existing methods struggle to adapt to evolving research needs. This study synthesizes prior research on LLMs in medical exams, highlighting current limitations and proposing future research directions.

**Methods and analysis:**

The formulation of the protocol was guided by the standards set forth in the *JBI Manual for Evidence Synthesis*. Following the establishment of precise inclusion/exclusion criteria and search strategies, we will execute systematic searches in the PubMed and Web of Science Core Collection databases. The method encompasses literature review, data extraction, analytical frameworks, and process mapping. By employing this method, researchers maintain methodological rigor during the entire research process.

**Ethics and dissemination:**

This protocol describes a method for performing a scoping review. The investigation focuses on the organized synthesis and examination of previously published research. It does not include human/animal experimentation or sensitive data collection. Ethical approval is not required for this literature-based study.

## Introduction

The rapid advancement of Artificial Intelligence (AI), particularly Large Language Models (LLMs), has created new opportunities in medical education and assessment. By leveraging deep learning and large datasets, LLMs can generate, understand, and analyze natural language, demonstrating cognitive reasoning and language comprehension approaching human levels. They have proven effective across multiple professional fields, including medicine, law, and business [1], supporting the acquisition of specialized knowledge and training in analytical tools [2]. Their application in medical education appears particularly promising.

Medical examinations are essential in medical education for assessing students’ readiness for clinical practice. Researchers now use standardized assessments, including multilingual and multiregion medical licensing exams, to evaluate LLMs’ ability to apply medical knowledge. The proficiency of LLMs in solving complex questions underscores their potential as instructional tools in medical education.

For instance, OpenAI’s ChatGPT, based on GPT-3.5 and GPT-4 [3], has performed strongly in several examinations, including the Neurosurgery Written Board Examinations [4], the UK Radiology Fellowship Examinations [5], and the Dental Licensing Examinations [6]. Both models surpassed passing thresholds, with GPT-4 showing superior performance. Its enhancements notably improved accuracy on factual and reasoning questions, approaching human-level performance across academic and professional contexts [7].

Similarly, Google’s Bard (now Gemini), based on the PaLM2 architecture [3]. surpassed passing thresholds in several standardized examinations, including the Family Medicine In-Training Exam [8], the Ophthalmology Knowledge Assessment [9], and the Japanese national dental hygienist examination [10].

Bing Chat (now Microsoft Copilot), based on the GPT-4 architecture, has performed well on medical assessments such as the Korean Emergency Medicine Board Examination [11] and the Peruvian National Licensing Medical Examination [12]. LLMs’ achievements extend beyond English-language tests, demonstrating effectiveness across languages, regions, and specialties, including the Spanish Medical Residency Entrance Examination (MIR) [13] and the Intercollegiate Membership of the Royal College of Surgeons examination [14].

Research indicates that while LLMs generally perform well, they often underperform in niche specialties. On the Japanese Society of Radiology Official Board Exam [15], ChatGPT scored 40.8%, GPT-4 65%, and Google Bard 38.8%. On the American Board of Anesthesiology (ABA) Examination [16], only GPT-4 passed, achieving 78% on the basic and 80% on the advanced sections, whereas GPT-3 scored 58% and 50%, and Google Bard 47% and 46%, respectively.

LLMs perform well on fill-in-the-blank tasks, short-answer questions, and expository prompts [17] and can accurately respond to questions based on articles or charts. However, their performance declines on tasks requiring analog data interpretation, detailed written explanations, or complex problem-solving [17]. Differences in national healthcare regulations, policies, and languages further create training gaps, favoring English-language contexts.

A standardized medical examination is an assessment where the procedures, administration, materials, and scoring rules are fixed so that as far as possible the assessment is the same at different times and places [18]. In this study, the Medical Postgraduate Entrance Examination and the Medical Student Entrance Examination are considered standardized examinations, as they are conducted under uniform conditions, follow fixed procedures, have clear assessment objectives, and apply standardized scoring criteria. The two examinations differ in focus: the former emphasizes advanced cognitive abilities and comprehensive medical knowledge, while the latter targets foundational knowledge, cognitive skills, and academic potential. Nonetheless, both maintain high standardization and yield comparable results.

Additionally, this study includes several standardized medical assessment tasks, such as specialty self-assessment programs and differential diagnosis exercises. These tasks follow clear educational or clinical frameworks and provide expert-validated answers, allowing for standardized evaluation of LLM performance on medical examinations. Based on this, the study establishes a framework for evaluating LLMs, and the scoping review addresses the following core questions(see Table 1). The detailed research design is provided in the Discussion section.

**Table 1:** Research sub-questions to be answered based on the scoping review.

RQ.01-Modelling and training	Which LLMs are used and how does the pre-training process work?
RQ.02-Use Cases	Which question types and which specialties do medical examinations correspond to?
RQ.03-Data and notes	How much data was used for model training, how was the annotation process designed, and is the data publicly available?
RQ.04-Performances	How accurate are LLMs for medical examinations?
RQ.05-Analysed	What are the types of errors LLMs make on the examinations?
RQ.06-Challenge	What are the open challenges and common limitations of existing LLMs?
LLMs, Large Language Models	

## Methods and analysis

A scoping review systematically maps existing evidence through comprehensive literature searching, evaluation, and screening. It identifies core principles, types of evidence, and research gaps within defined domains. This review follows the *JBI Manual for Evidence Synthesis* (Chapter 11: Scoping Reviews) and adheres to the PRISMA-ScR guidelines [19] (S1 File). This protocol excludes research involving human or animal subjects and related data, thereby avoiding ethical approval requirements. Its methodology specifies inclusion and exclusion criteria, search strategy, sources of evidence, data extraction, analysis, and result presentation, as detailed in subsequent chapters. Immediately following the announcement of this scoping review, work will commence, with final findings to be published in open-access peer-reviewed medical education journals and any protocol changes documented along with their dates and reasons.

### Inclusion exclusion criteria

In Table 2 and Table 3. This section defines the literature selection criteria, aligning them with the scoping review’s title, primary research questions, and sub-questions.

**Table 2:** Inclusion criteria.

1.	The scope of the study is medical examinations. (Includes final exams, licensing exams, physician licensing tests, medical school entrance exams, etc.)
2.	The purpose of the study was to summarize the performance of LLMs in medical examinations in the medical field.
3.	The included studies comprised formally published, peer-reviewed journal articles, conference papers, and letters.
4.	An article on the performance of LLMs in arbitrary languages.
5.	Literature on different versions and types of large models.
6.	Topics include, but are not limited to, multiple-choice, open-ended questions on literature in the field of medical examinations questions.

**Table 3:** Exclusion criteria.

1.	Literature in the field of non-medical examinations.
2.	Studies not related to LLMs.
3.	Repeat study.

### Search strategy

This study examinations the application and evaluation of LLMs in medical examinations and educational assessment. PubMed and the Web of Science Core Collection were chosen as primary databases, as they are authoritative sources in the medical and biomedical fields and systematically index peer-reviewed literature. This selection ensures reproducibility, comparability, and methodological consistency. The search strategy was implemented in three steps: first, preliminary searches in both databases identified key terms for query development (Table 4), with the full search formula provided in the supporting information (S2 File); second, systematic search queries were iteratively refined based on these terms.

**Table 4:** Primary search terms.

PubMed	((((((((((AI (Artificial Intelligence))[MeSH Terms]) OR (ChatGPT[Title/Abstract])) OR (LLM[Title/Abstract]))) AND (Medicine[MeSH Terms])) OR (medical[Title/Abstract])) OR (Traditional Chinese Medicine[Title/Abstract])) AND (Educational Measurement[MeSH Terms])) OR (licensing examination[Title/Abstract])) OR (Graduate Records Examination[Title/Abstract])
Web of Science Core Collection	((TS=(Large language models)) AND (TS=(Medical Tests))

This review focuses on publications from January 1, 2023, to December 31, 2025, a three-year period selected to capture the most recent and representative advances in LLM applications in medical examinations while minimizing timeliness bias. Given the rapid pace of technological development in medical AI, focusing on recent literature provides a more accurate reflection of current research trends. Earlier work (e.g., 2020–2022), including exploratory studies such as early GPT-3 investigations, was limited and may not be fully captured by our search strategy. Nonetheless, the selected timeframe sufficiently represents the core developments and evolution of this field.

After selecting studies, researchers will perform forward citation searches (SOE) on reference lists to ensure comprehensive topic coverage. This approach identifies relevant studies missed in preliminary searches. Reviewing references from selected articles will uncover theme-related literature and expand the research scope.

### Source of evidence

**Fig 1:**
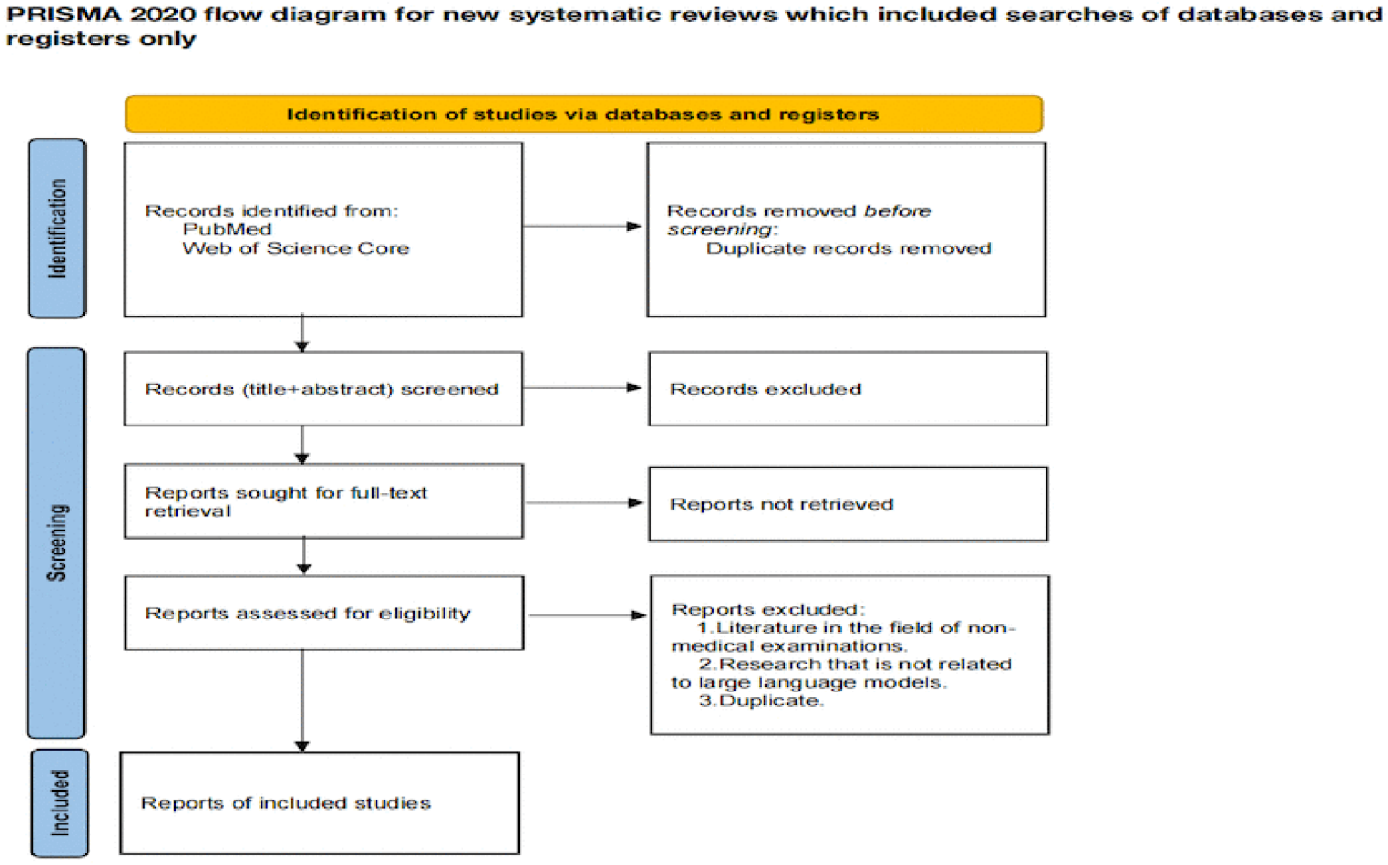
Evidence source selection process.

Three reviewers independently screened the retrieved literature. Inter-reviewer agreement was assessed using the pairwise Cohen’s kappa coefficient, which accounts for chance agreement and is more robust than simple percent agreement. Disagreements or kappa values below the predefined threshold were resolved by the corresponding author, who made the final inclusion decision. Screening followed predefined criteria: after applying the search strategy and removing duplicates, titles and abstracts were screened, followed by full-text assessment. Any remaining discrepancies were adjudicated by a fourth reviewer, and studies not meeting eligibility criteria were excluded. Fig 1 illustrates the process described.

### Data extraction

We will systematically extract key information from selected literature using data extraction forms based on the *JBI Evidence Synthesis Manual*, customized to align with the specific objectives and subfields of the review (see Box 1). Prior to the full study, we conducted a pilot test on 20 selected articles to validate the data extraction table. The results indicated that no modifications to the fields were necessary, allowing direct extraction of data for the six core research questions. During the pilot phase, reviewers assessed and reached consensus using Cohen’s kappa, calculated as follows:


$$\kappa =\frac{P_{\text{0}}-P_{e}}{\text{1}-P_{e}}$$

Here, $P_{\text{0}}$ represents the observed proportion of agreement, and $P_{e}$denotes the expected proportion of agreement by chance. The tested forms will be uploaded as supporting information (S3 File).

**Box 1. Data charts.**

**Table:** 

**Scope review details** · Title · DOI · Main issues and sub-issues of the study
**Details extracted from sources of evidence (divided into different questions)** **Literature** · Year of publication · Article Overview · Purpose of the article · Conclusion of the article · Number of included studies · Research method
**Model Characteristics** · Category of models used · Description of the application of the model · Model accuracy **Examination Type** **·** Type/scope of examination **·** Examination Question Types

### Analysis of evidence and presentation of results

This study employs descriptive analysis to categorize evidence and provide a comprehensive overview of current research. LLMs perforance in medical examinations was analyzed using data visualization, with percentages as the primary metric, highlighting performance differences across examinations and supporting exploration of sub-questions (see Table 1). Detailed graph captions facilitate precise interpretation, clarify significance, and enable standardized analysis. During data extraction, content from the original texts was entered according to predefined fields, with missing information marked as ‘/’. To ensure consistency and comparability, a multi-dimensional evaluation was applied. LLMs performance were categorized rather than directly compared to actual scores: 80%–100% accuracy was rated excellent, 60%–80% good, and below 60% poor. This study is qualitative rather than quantitative.

## Discussion

LLMs have demonstrated promising applications in medical examinations. The complete study will be presented in six detailed parts: (1) Medical specialties and test question types covered by LLM applications; (2) Types of LLMs used and iteration trends; (3) Data volume and availability for LLM training; (4) Performance of LLMs in medical examinations. Future research will advance in four phases: first, comparing LLMs performance against human physicians/students; second, evaluating cross-language performance by translating test questions into English and assessing original-language versions; third, assessing performance differences across model versions; fourth, analyzing performance evolution during LLM development, with a focus on text comprehension and multimodal capabilities; (5) Categorize LLM response errors, including non-linguistic comprehension barriers, insufficient multimodal capabilities, inconsistent answers, and hallucinations, with future refinements in classification; (6) Identify challenges and limitations of LLMs in medical exams, offering guidance for meeting the growing demands for safety, interpretability, and robustness in complex medical applications.

Based on this research framework, this study offers several advantages: (1)This study systematically compares iterative LLM versions using multidimensional human–model and model–model evaluations of medical examination performance. (2)Compared with studies focusing on a single metric, this study analyzes LLMs across six dimensions, including pre-training characteristics, accuracy, and error types, to provide a more comprehensive performance assessment. Nevertheless, this study has several limitations: (1)The literature search was limited to PubMed and the Web of Science Core Collection database, which may have led to missing relevant studies. (2)Reliance on publicly available pre-training data and limited high-quality annotated medical datasets may constrain LLM performance. (3)The predominance of English-language medical examinations may introduce language bias in non-English applications.

## Supporting information

S1 File PRISMA-P checklist. (DOCX)

S2 File Supplementary material searchable. (DOCX)

S3 File Pilot testing of data extraction form. (XLSX)
