# Supplementary material for: Evaluation of large language models in medical examinations: A scoping review protocol
Source: PLoS One. 2026 Apr 22;21(4):e0347539. doi: 10.1371/journal.pone.0347539 (PMC13102214; doi:10.1371/journal.pone.0347539)
Supplement: S2 File — (DOCX) [file pone.0347539.s002.docx]

**Attachment**

**Search Formula**

**PubMed**：(((((((((((((("Artificial Intelligence"[Mesh]) OR (Artificial Intelligence[Title/Abstract])) OR (Computer Reasoning[Title/Abstract])) OR (AI (Artificial Intelligence[Title/Abstract]))) OR (Machine Intelligence[Title/Abstract])) OR (Computational Intelligence[Title/Abstract])) OR (ChatGPT[Title/Abstract])) OR (LLM[Title/Abstract])) OR (GPT[Title/Abstract])) OR (ERNIE Bot[Title/Abstract])) OR (ChatGLM[Title/Abstract])) AND ((((((((("Medicine"[Mesh]) OR (Medicine[Title/Abstract])) OR (Medical Specialties[Title/Abstract])) OR (Medical Specialty[Title/Abstract])) OR (Medical Speciality[Title/Abstract])) OR (Medical Specialities[Title/Abstract])) OR (nursing[Title/Abstract])) OR (medical[Title/Abstract])) OR (Traditional Chinese Medicine[Title/Abstract]))) AND ((((((((((((((("Educational Measurement"[Mesh]) OR (Educational Measurement[Title/Abstract])) OR (Educational Measurements[Title/Abstract])) OR (Graduate Records Examination[Title/Abstract])) OR (Graduate Records Examinations[Title/Abstract])) OR (Occupational Therapy[Title/Abstract])) OR (licensing examination[Title/Abstract])) OR (medical licensing examination[Title/Abstract])) OR (biomedical science exams[Title/Abstract])) OR (Medical Residency Examination[Title/Abstract])) OR (dental hygienist examination[Title/Abstract])) OR (Nursing Licensure Examinations[Title/Abstract])) OR (standardized examinations[Title/Abstract])) OR (Pharmacy licensure[Title/Abstract])) OR (NMLE[Title/Abstract]))))

**Web of Science**：((((TS=(Large language models)) OR TS=(ChatGPT)) AND TS=(Medical Tests)) AND TS=(Medical Examination))
